# The inequalities and determinants of Households’ Distress Financing on Out-off-Pocket Health expenditure in Malaysia

**DOI:** 10.1186/s12889-022-12834-5

**Published:** 2022-03-07

**Authors:** Nor Zam Azihan Mohd Hassan, Mohd Shaiful Jefri Mohd Nor Sham Kunusagaran, Nur Amalina Zaimi, Farhana Aminuddin, Fathullah Iqbal Ab Rahim, Suhana Jawahir, Zulkefly Abdul Karim

**Affiliations:** 1 Centre of Health Economics Research (CHEeR), Institute for Health Systems Research (IHSR), Ministry of Health Malaysia, Kompleks Institut Kesihatan Negara (NIH), Blok B2, No.1, Jalan Setia Murni U13/52, Seksyen 13 Setia Alam, 40170 Shah Alam, Selangor Darul Ehsan, Malaysia; 2 Faculty of Economics and Management, Center for Sustainable and Inclusive Development (SID), Universiti Kebangsaan Malaysia (UKM), Bangi, Malaysia

**Keywords:** Distress financing, Inequality, Determinants, Household, Healthcare

## Abstract

**Background:**

Out-of-pocket (OOP) payments for healthcare services potentially have severe consequences on households, especially among the poor. Under certain circumstances, healthcare payments are financed through selling household assets, or borrowings. This certainly could influence households’ decision, which likely resorts to forgoing healthcare services. Thus, the focal point of this study is aimed to identify the inequalities and determinants of distress financing among households in Malaysia.

**Methods:**

This study used secondary data from the National Health and Morbidity Survey (NHMS) 2019, a national cross-sectional household survey that used a two-stage stratified random sampling design involving 5,146 households. The concentration curve and concentration index were used to determine the economic inequalities in distress financing. Whereas, the determinants of distress financing were identified using the modified Poisson regression model.

**Results:**

The prevalence of borrowing without interest was the highest (13.86%), followed by borrowing with interest (1.03%) while selling off assets was the lowest (0.87%). Borrowing without interest was highest among rural (16.21%) and poor economic status (23.34%). The distribution of distress financing was higher among the poor, with a concentration index of -0.245. The modified Poisson regression analysis revealed that the poor, middle, rich, and richest had 0.57, 0.58, 0.40 and 0.36 times the risk to develop distress financing than the poorest socio-economic group. Whereas, the presence of one and two or more elderly were associated with a 1.94 and 1.59 times risk of experiencing distress financing than households with no elderly members. The risk of developing distress financing was also 1.28 and 1.58 times higher among households with one and two members receiving inpatient care in the past 12 months compared to none.

**Conclusions:**

The findings implied that the improvement of health coverage should be emphasized to curtail the prevalence of distress financing, especially among those caring for the elderly, requiring admission to hospitals, and poor socio-economic groups. This study could be of interest to policymakers to help achieve and sustain health coverage for all.

## Introduction

Households are subjected to out-of-pocket (OOP) health spending when they use or receive any health care services. These include promotive, preventive, curative, rehabilitative, palliative (or long-term), or laboratory services [1]. OOP payments for healthcare services can potentially lead to adverse consequences on the patients and their families, especially in the absence of health protection mechanisms. High OOP health spending is often associated with increased poverty and mental health problems such as depression, anxiety and stress [2, 3]. Without adequate cash in hand, people are constrained to various methods to finance healthcare, such as selling assets, borrowing, reducing foods consumption, withdrawing children from schools, and even foregoing or delaying seeking medical care. [4, 5].

A distress or hardship health financing occurs when someone turns to either borrowing or selling assets to finance their healthcare expenses [6]. High OOP health expenditure, low insurance coverage as well as limited government expenditure on health are some of the causes of distress financing [7]. Studies among low- and middle-income countries found that borrowing or selling assets occurs at an average of 22% and 10%, respectively [8]. In desperate situations, people can resort to borrowing with or without interest taking into consideration their socio-economic status, repayment period, and the nature of the loan [9]. According to several studies done in the low- and middle-income countries, familiar sources of distress financing can be in the form of borrowing with or without interest, either from a financial institution, friends, or family members, selling assets such as crops and livestock as well as mortgaging assets [10, 11]. Out of these, borrowing with interest tends to cause tremendous economic hardship than borrowing without interest due to the large sum of money they need to repay. [12]. Prior studies conducted in Argentina, India, Tanzania and rural China found that distress financing was unequally distributed and mainly affected the poor [9, 13].

Apart from poor socio-economic status, indebtedness may also have detrimental effects on physical and mental health. This frequently leads to worsening financial problems, thus creating a vicious cycle of health demands and indebtedness. The reason could conceivably be the additional healthcare needs as well as the inability to work and gain income. Studies found that indebtedness and the failure to repay loans can lead to depression, stress, and poor health [14–16]. Stress was found to influence health-related behaviours and cause psychological changes, which are significant in various disease processes, such as cardiovascular and metabolic diseases [17, 18]. High-interest borrowing and debt can also have a multiplier effect on health, especially among those unable to repay their debt [15].

Studies on the determinants of distress health financing were relatively limited. Past studies found that distress financing is determined by the household’s socio-economic status, household size, and the use of inpatient and outpatient care, especially in the private health sectors [19]. Households in the lower socio-economic group tend to resort to borrowing and often end up unable to pay off their debt [19]. Besides, borrowing was also common among those with bigger household sizes and higher health expenses [8]. Distress financing also was more common among those admitted to the hospital, having to care for their elderly parent, seeking maternity care, using private health care facilities, and having family members with non-communicable diseases (NCDs) [6, 20]. Studies also found that those living in urban areas tend to have minimal risk of financial distress due to the widely available healthcare services [21]. In addition, the availability of medical protection plans and initiatives such as private insurance, government guarantee letters, panel clinics, and others seem to reduce the incidence of distress financing among households [22].

World Health Organization (WHO) has highlighted the importance of the country’s healthcare systems to provide essential health services to all without financial hardship through Universal Health Coverage (UHC) [23]. Nevertheless, many countries still opted for the least sustainable option to finance healthcare through the OOP mechanism [23]. This was made worse by the never-ending issue of equity in health service delivery [24]. This includes the issue of inequality in distress health financing among households, which could also hinder the country’s effort in achieving UHC. The term ‘inequalities’ often describes the variation of health status among different socio-economic statuses, geographic locations, employment status, gender and ethnicity [25]. The health financing system of a country among others would set the provision of health service delivery and thus, become the main factor in making sure equality in population health status. There are various methods for measuring inequalities such as concentration curve, concentration index, Gini coefficients, and others. The concentration index measures horizontal health inequalities based on socio-economic status and is calculated from the concentration curve by quantifying the disparity of one variable against the distribution of another selected variable [26]. It is one of the common measures of socio-economic health-related inequalities [27, 28].

The issue of inequality is not limited to one but all countries including Malaysia. The two-tier healthcare system in Malaysia is provided by both the highly subsidised public sector and pay-for-service in the private service sector. The Ministry of Health only recouped 2–3% of the total patient charges. While the health services such as hospital admission in the public sector only require patients to bear the minimal cost, it acts as the safety net by ensuring minimal possible financial risk to access health services [29]. Hence, patients who could not afford private healthcare charges will opt for public healthcare services. Overall, the high utilization of health services in Malaysia was equally distributed across socio-economic groups. Studies found that the use of private health services in Malaysia increases with household income, while the use of public health services is more pro-poor [30]. The latest data showed that outpatient healthcare services in Malaysia are composed of 64.3% public and 35.7% private sector [31]. Whereas, public health sectors contribute about 75.5% and 79.5% to inpatient and oral health care, respectively [31]. Medical private insurance in Malaysia is voluntary and only covers health expenses in private health sectors. Other medical protection programmes include, but are not limited to; (1) Social Security Organisation (SOCSO) and Employee Provident Funds (EPF), which are two social security funds in Malaysia that provide health coverage for employees working in the private sector; (2) employer insurance scheme, which is provided by some employers in private sectors to their employees; and (3) guarantee letters for the government servants, which allows free health provision among the government servants [32]. Other health financing initiatives provided by the governments for accessing healthcare services include those catered for the bottom 40% of population (B40) groups such as *Skim Peduli Kesihatan* for the B40 (PeKa B40) and mySalam [33].

Despite this, the Malaysia National Health Account (MNHA) reported that Malaysia’s OOP expenditure remains high at around 30–40% of total health expenditure [34]. This is well beyond WHO’s suggestion of 15–20% [35, 36]. This is also comparatively higher than most OECD countries [37]. However, high OOP expenses are more prevalent among households in high socio-economic groups [37]. While the OOP expenditure did not show a reduction trend albeit higher cost of care over the years, concerns were raised about the sustainability of the government to continue with the current health financing system and the need for healthcare reforms [34]. Despite that, the incidence of catastrophic health expenditure (CHE) in Malaysia was relatively low. The Malaysia Health Care Demand Analysis using 2009/10 data reported that the incidence of households spending more than 10% of the total household expenditure was around 1.44% [38]. In comparison, the incidence of households spending more than 25% of household expenditure was around 0.16%. This is relatively low compared to the neighbouring countries such as Thailand, Vietnam, Indonesia and others. Overall, the financial risk protection in Malaysia has improved over the last decades with the prevalence dropping more than 50% [38].

It is also suggested that 5–6% of Gross Domestic Product (GDP) is required to provide UHC assuming a single public financing health system [39, 40]. Hence, Malaysia’s government spending at around 2% of GDP provides additional challenges to achieving equal access to healthcare services. Nevertheless, a report showed that the Kakwani’s progressivity index for the tax-financed system in Malaysia was slightly progressive with an index value of 0.186. The progressive finance sources include direct taxes, private insurance, OOP and contributions to EPF and SOCSO [41].

Given this backdrop, the present study contributes to two dimensions. First, to the policymakers and stakeholders by providing guides on the current achievement of UHC and a piece of evidence on the effectiveness of financing health systems in Malaysia. Secondly, this study also contributes to the literature on OOP expenditure by extending the current knowledge on the determinants of household distress financing and their level of inequalities in middle-income countries, especially in Malaysia.

There are relatively no documented studies exploring distress health financing in terms of the determinants and inequality in Malaysia. This kind of study is beneficial to the decision-makers as a guide to improving the health financing systems in Malaysia. Hence, this study aims to identify inequalities and determinants of distress financing among the households in Malaysia to provide a better understanding especially to the policymakers for a better improvement of UHC in Malaysia.

## Methods

### Data sources

This study used cross-sectional datasets from the National Health and Morbidity Survey (NHMS) 2019, a national household survey that targeted all non-institutionalised populations residing in Malaysia. The NHMS (2019) sampled a cross-section of households in all 13 states and three federal territories in Malaysia using a two-stage proportionate to size cluster sampling to achieve national representativeness. The stratification was performed by states and federal territories constituting the primary stratum, followed by urban and rural within the primary stratum as the secondary stratum [31]. Department of Statistics Malaysia (DOSM) defines urban as “any gazetted areas plus their adjoining built-up areas, of which the combined population is at least 10,000 during census 2010 or any special development areas with a population of more than 10,000, of which at least 60% (15 years and above) involved in non-agricultural activities” [42].

First, all Enumeration Blocks (EBs) were randomly selected by the probability proportional to the size sampling method. A total of 350 EBs were selected for urban areas, and 113 EBs were for rural areas, yielding a total of 463 EBs. Subsequently, 14 Living Quarters (LQs) were randomly selected from each selected EB. This random selection of EBs and LQs was performed and provided by the DOSM. All households within the selected LQs were included. In this study, a household is defined as individuals living together in the same house and having common arrangements for basic domestic activities such as cooking and eating.

### Data collection

Data were collected from July to October 2019. A face-to-face interview was conducted by the trained personnel using a bilingual (Malay and English), structured, and validated questionnaire [43, 44]. NHMS 2019 official report described the methodology and sampling in detail [31]. A total of 5,206 households were interviewed (3,196 in urban and 1,950 in rural). Missing data were handled by the listwise deletion method. Since this study has a large sample size and the data is Missing Completely at Random (MCAR), the power is not an issue, thus listwise deletion is a reasonable strategy to be adopted [45, 46]. After removing missing data, 5,146 households were included in the analysis.

### Variables Descriptions

#### Dependent variable

The dependent variable of interest used in this study was distress financing. It was a binary variable that shows the likelihood of a household using sales of assets, borrowing without interest (from family and friends), and borrowing with interest as a source of financing.

#### Explanatory variables

**Fig. 1:**
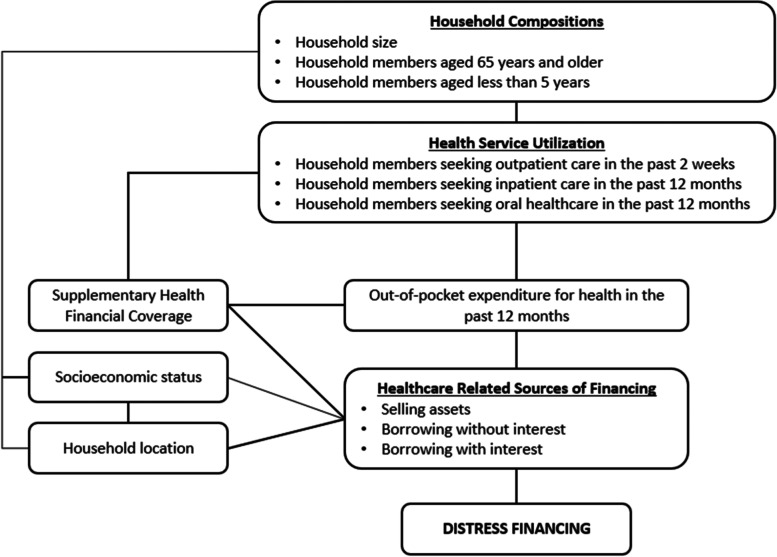
Explanatory variables associated with distress financing (adapted from Ir et al.) [19]

Several explanatory variables were identified for the analysis (Fig. 1). Household demographic characteristics included were household location (urban, rural), a total of household members aged 65 years and older (0, 1, 2, more than 2), a total of household members aged less than five years (0, 1, 2, more than 2) and household size (less than 5, 5 or more). The socio-economic status of the household was calculated based on monthly household income, ranked, and classified into a wealth quintile of poorest (1st quintile), poor (2nd quintile), middle (3rd quintile), richer (4th quintile), and richest (5th quintile). The explanatory variables used to measure health service utilisation were the total of household members who received outpatient care in the past two weeks, the total of household members who received inpatient care in the past 12 months, and the total of household members who received oral healthcare in the past 12 months. Supplementary health coverage refers to the number of supplementary financial health coverage received by the households or household members seeking health services (no health coverage, one health coverage, more than one health coverage). In Malaysia, these supplementary health coverages include government guarantee letters, private insurance, employer insurance, SOCSO, and others. The OOP expenditure for health in the past 12 months was calculated based on the cumulative self-reported OOP spending for each household member. Household OOP expenditures were grouped into five categories, namely less than Malaysian Ringgit (MYR) 100, MYR 100 to < 200, MYR 200 to < 300, MYR 300 to < 400, and MYR 400 and more, wherein MYR 1.00 ~ USD 0.24 [47].

### Statistical analysis

The data were analysed using Stata software version 14 (Stata Corp, College Station, Texas, USA) and Microsoft Excel 2016. Initial descriptive statistics were performed to explain the characteristics of households with distress financing. Subsequently, analysis was conducted to determine the prevalence of distress financing among different household locations and socio-economic statuses. The prevalence of households having distress financing was measured by dividing the total number of households with distress financing by the total number of households [48].

Concentration curve and concentration index were often used for determining the economic inequalities in distress financing. This study used both methods to estimate the economic inequality in distress financing. The concentration curve of distress financing and various sources of financing were visualised by a graphical representation of the cumulative proportion of distress sources of financing (y-axis) against the cumulative proportions of the population ranked by the household’s socio-economic status (x-axis). The concentration curve will coincide with the line of equality if distress financing or the various sources of financing is evenly distributed across the socio-economic status. However, if the concentration of distress financing belongs to the higher (lower) socio-economic status, the concentration curve would fall below (above) the line of equality [28]. The concentration index is derived from the concentration curve and calculated as twice the area between the equality line and the concentration curve. Concentration index value ranges from − 1 to + 1. The negative value signifies the distress financing is concentrating on the poor, while, the positive value reflects concentration among the rich. The zero concentration index indicates no inequality in the distribution of distress financing.

The concentration index, *C* can be measured using the following formula [49]:

1 $$C=\left({p}_{1}{L}_{2}-{p}_{2}{L}_{1}\right)+\left({p}_{2}{L}_{3}-{p}_{3}{L}_{2}\right)+\dots +\left({p}_{T-1}{L}_{T}-{p}_{T}{L}_{T-1}\right)$$

In Eq. (1), *p*_*T*_ is the cumulative proportion of the sample ranked by socio-economic status in group *T*, and *L*_*T*_ is the corresponding cumulative proportion of the health variables, which is the distress health financing and the various sources of financing. In this study, the concentration indices were calculated using the CONCINDC module in Stata software [50, 51].

The determining factors of distress financing were identified using bivariate and multivariate statistical analysis. Potential variables were initially identified through bivariate analysis with explanatory variables of *p*-value < 0.25 were incorporated into the modified (or robust) Poisson regression model. The modified Poisson model was generally preferable because it provided unbiased estimates of relative risks (or risk ratios) especially in dealing with model misspecification [52]. Unlike the log-binomial model, the modified Poisson model does not frequently result in a non-convergence problem. The robust variance estimation (or classical sandwich estimator) used could prevent over-estimation of the standard errors of parameters estimates [53].

The mathematical model of the study is written as the following:

2 $$log\left(E\left({Y}_{i}\right)\right)={\beta }_{0}+{\beta }_{1}{X}_{1i}+{\beta }_{2}{X}_{2i}+{\beta }_{3}{X}_{3i}+{\beta }_{4}{X}_{4i}+{\beta }_{5}{X}_{5i}+{\beta }_{6}{X}_{6i}+{\beta }_{7}{X}_{7i}+{\beta }_{8}{X}_{8i}+{\beta }_{9}{X}_{9i}+{\beta }_{10}{X}_{10i}+{u}_{i}$$

In Eq. (2), *Y*_*i*_ is the binary outcome of having distress financing. *X*_*1*_ refers to household location, *X*_*2*_ is the total of household members aged 65 years and older, *X*_*3*_ is the total of household members aged less than five years, *X*_*4*_ is household size, *X*_*5*_ is the socio-economic status, *X*_*6*_ is the total of household members received outpatient care in the past two weeks, *X*_*7*_ is the total of household members received inpatient care in the past 12 months, *X*_*8*_ is the total of household members received oral healthcare in the past 12 months, *X*_*9*_ is the supplementary health coverage, *X*_*10*_ is the OOP expenditure for health in the past 12 months, *u*_*i*_ is the random error term, *i* is the household (cross-section), and *β* is a beta coefficient to be estimated. The Eq. (2) can also be expressed as below:

3 $$E\left({Y}_{i}\right)={e}^{{\beta }_{0}+{\beta }_{1}{X}_{1i}+{\beta }_{2}{X}_{2i}+{\beta }_{3}{X}_{3i}+{\beta }_{4}{X}_{4i}+{\beta }_{5}{X}_{5i}+{\beta }_{6}{X}_{6i}+{\beta }_{7}{X}_{7i}+{\beta }_{8}{X}_{8i}+{\beta }_{9}{X}_{9i}+{\beta }_{10}{X}_{10i}+{u}_{i}}$$

The Relative Risk (RR) is given by ${e}^{\beta }$ as shown in Eq. (3).

The modified Poisson regression model adopts a classical sandwich estimator under the generalized estimation equation (GEE) framework in providing accurate standard errors for the parameter estimates [54]. The variance-covariance matrix can be explained by the following:

4 $${\left[\sum _{i = 1}^{n}E\left[{I}_{i}\left(\beta \right)\right]\right]}^{-1}{\left[\sum _{i = 1}^{n}E\left[\left({S}_{i}\left(\beta \right){S}_{i}{\left(\beta \right)}^{T}\right)\right]\right]}^{-1}{\left[\sum _{n = 1}^{n}E\left[{I}_{i}\left(\beta \right)\right]\right]}^{-1}$$

Where ${I}_{i}\left(\beta \right)=-\frac{\partial {S}_{i}\left(\beta \right)}{\partial \beta }$ denotes information matrix. By evaluating the variance-covariance matrix at $\widehat{\beta }$, a consistent estimate of the variance can be achieved.

For the modified Poisson regression model, a *p*-value of less than 0.05 is considered statistically significant.

### Ethics

NHMS 2019 follows the tenets of the Declaration of Helsinki throughout the conduct of the study. Before the interview, written informed consent was obtained from all participants. Ethics clearance was obtained from Medical Research and Ethics Committee (MREC), Ministry of Health Malaysia (MOH) Malaysia, and registered under National Medical Research Register, MOH Malaysia (NMRR-18-3085-44207).

## Results

### Prevalence of various sources of financing

Table 1 shows the prevalence of various sources of distress financing by household location and socio-economic status in Malaysia. The results showed that the prevalence of borrowing without interest was the highest at 13.86%, followed by borrowing with interest (1.03%) while selling off assets was the lowest at 0.87%. The prevalence of borrowing without interest was higher among households living in the rural area (16.21%) while the sale of assets was higher among households living in the urban area (0.97%). The prevalence of borrowing with interest was similar among households living in urban and rural areas at 1.03%.

In terms of socio-economic status, households in the middle (1.06%) and poorest (1.05%) economic groups have the highest prevalence of selling assets. Otherwise, households in rich and poor economic groups have the lowest selling assets with 0.69% and 0.73%, respectively. The prevalence of borrowing without interest was the highest among households in the poorest economic status at 25.54%. In comparison, households in the rich and the richest economic groups have the lowest prevalence at 7.44% and 6.19%, respectively. The results also showed that borrowing with interest was the highest among households in the middle economic group (1.59%) and the lowest among households in the poorest economic group (0.56%).

**Table 1:** Prevalence of Various Sources of Distress Financing by household location and socio-economic status in Malaysia (*n* = 5,146)

Characteristics	n	Sell of assets		Borrowing without interest		Borrowing with interest	
		I (%)	95% CI	I (%)	95% CI	I (%)	95% CI
**Household location**							
Urban	3,196	0.97	0.68, 1.38	12.42	11.32, 13.61	1.03	0.73, 1.45
Rural	1,950	0.72	0.43, 1.21	16.21	14.63, 17.91	1.03	0.66, 1.58
**Socio-economic status**							
Poorest	1,425	1.05	0.64, 1.74	25.54	23.34, 27.87	0.56	0.28, 1.12
Poor	1,098	0.73	0.36, 1.45	12.20	10.40, 14.28	0.73	0.36, 1.45
Middle	941	1.06	0.57, 1.96	10.63	8.81, 12.76	1.59	0.96, 2.63
Rich	874	0.69	0.31, 1.52	7.44	5.87, 9.38	1.26	0.70, 2.26
Richest	808	0.74	0.33, 1.64	6.19	4.72, 8.07	1.36	0.76, 2.44
**Total**	5,146	0.87	0.65, 1.17	13.86	12.94, 14.83	1.03	0.79, 1.35

Note: I is Prevalence (%), and CI is the Confidence Interval

### Household Characteristics

Table 2 demonstrates the households characteristics and the relationship of distress financing between the variables. About 62.1% of households lived in the urban area, while the remaining 37.9% lived in the rural area. Of the total number of households, about 25.1% have at least one family member aged 65 years and older. The percentage of households with at least family members aged less than five years was about 18.7%. About 79.3% have a household size of less than five, while the remaining 20.7% have a household size of five or more. In terms of socio-economic status, about 27.7% of households were in the poorest socio-economic group. Whereas, only about 15.7% were in the richest socio-economic group.

Results also showed that about 24.7% of households had at least one member who visited outpatient care in the past two weeks. In comparison, only about 15.6% of households had at least one member who sought inpatient care in the past 12 months. The percentage of households that had at least one member received oral healthcare in the past 12 months was 44.8%. Also, about 67.4% of households had received at least one supplementary health financial coverage. About 84.5% of the households had OOP spending on health less than MYR 100 in the past 12 months, while only 9.3% of households spent OOP MYR 400 or more.

There were significant differences on distress financing according to household location (χ^2^ = 12.34, *p* < 0.001), total of household members 65 years and older (χ^2^ = 186.93, *p* < 0.001), total of household members aged less than five years (χ^2^ = 13.27, *p* = 0.001), household size (χ^2^ = 4.16, *p* = 0.041), socio-economic status (χ^2^ = 203.32, *p* < 0.001), total of household members received inpatient care in the past 12 months (χ^2^ = 10.32, *p* = 0.016), total of household members received oral healthcare in the past 12 months (χ^2^ = 18.66, *p* < 0.001) and supplementary health coverage (χ^2^ = 63.70, *p* < 0.001).

There was no significant difference between distress financing with the total of household members who received outpatient care in the past two weeks (χ^2^ = 4.89, *p* = 0.180) and OOP expenditure on health in the past 12 months (MYR) (χ^2^ = 2.88, *p* = 0.577).

**Table 2:** Characteristics of household by distress financing and the relationship of distress financing between the variables in Malaysia (*n* = 5,146)

Characteristics	n (%)	Distress Health Financing				*p*-value^a^
		**Yes**	**%**	**No**	**%**	
**Total**	5,146 (100.0)	774	15.0	4,372	85.0	**-**
**Household location**						< 0.001***
Urban	3,196 (62.1)	437	13.7	2,759	86.3	
Rural	1,950 (37.9)	337	17.3	1,613	82.7	
**Total of household members aged 65 years and older**						< 0.001***
0	3,853 (74.9)	429	11.1	3,424	88.9	
1	991 (19.2)	275	27.8	716	72.2	
2 and more	302 (5.9)	70	23.2	232	76.8	
**Total of household members aged less than 5 years**						0.001**
0	4,182 (81.3)	660	15.8	3,522	84.2	
1	736 (14.3)	78	10.6	658	89.4	
2 and more	228 (4.4)	36	15.8	192	84.2	
**Household size**						0.041*
Less than 5	4,081 (79.3)	635	15.6	3,446	84.4	
5 and more	1,065 (20.7)	139	13.1	926	86.9	
**Socio-economic status**						< 0.001***
Poorest	1,425 (27.7)	372	26.1	1,053	73.9	
Poor	1,098 (21.3)	144	13.1	954	86.9	
Middle	941 (18.3)	117	12.4	824	87.6	
Rich	874 (17.0)	77	8.8	797	91.2	
Richest	808 (15.7)	64	7.9	744	92.1	
**Total of household members received outpatient care in the past 2 weeks**						0.180
0	3,874 (75.3)	561	14.5	3,313	85.5	
1	1,005 (19.5)	170	16.9	835	83.1	
2	209 (4.1)	36	17.2	173	82.8	
More than 2	58 (1.1)	7	12.1	51	87.9	
**Total of household members received inpatient care in the past 12 months**						0.016*
0	4,345 (84.4)	625	14.4	3,720	85.6	
1	694 (13.5)	128	18.4	566	81.6	
2	97 (1.9)	20	20.6	77	79.4	
More than 2	10 (0.2)	1	10.0	9	90.0	
**Total of household members received oral healthcare in the past 12 months**						< 0.001***
0	2,840 (55.2)	475	16.7	2,365	83.3	
1	1,204 (23.4)	168	13.9	1,036	86.1	
2	611 (11.9)	63	10.3	548	89.7	
More than 2	491 (9.5)	68	13.8	423	86.2	
**Number of supplementary health coverage**						< 0.001***
No coverage	1,677 (32.6)	340	20.3	1,337	79.7	
One coverage	1,608 (31.2)	235	14.6	1,373	85.4	
More than 1 coverage	1,861 (36.2)	199	10.7	1,662	89.3	
**OOP expenditure for health in the past 12 months (MYR)**						0.577
Less than 100	4,347 (84.5)	653	15.0	3,694	85.0	
100 to < 200	195 (3.8)	25	12.8	170	87.2	
200 to < 300	77 (1.5)	9	11.7	68	88.3	
300 to < 400	47 (0.9)	6	12.8	41	87.2	
400 and more	480 (9.3)	81	16.9	399	83.1	

Note: ^a^ Chi Square Test

*p < 0.05, **p < 0.01, ***p < 0.001 (significance level at 10%, 5%, and 1%, respectively)

### Level of inequality for distress financing and various sources of financing

**Fig. 2:**
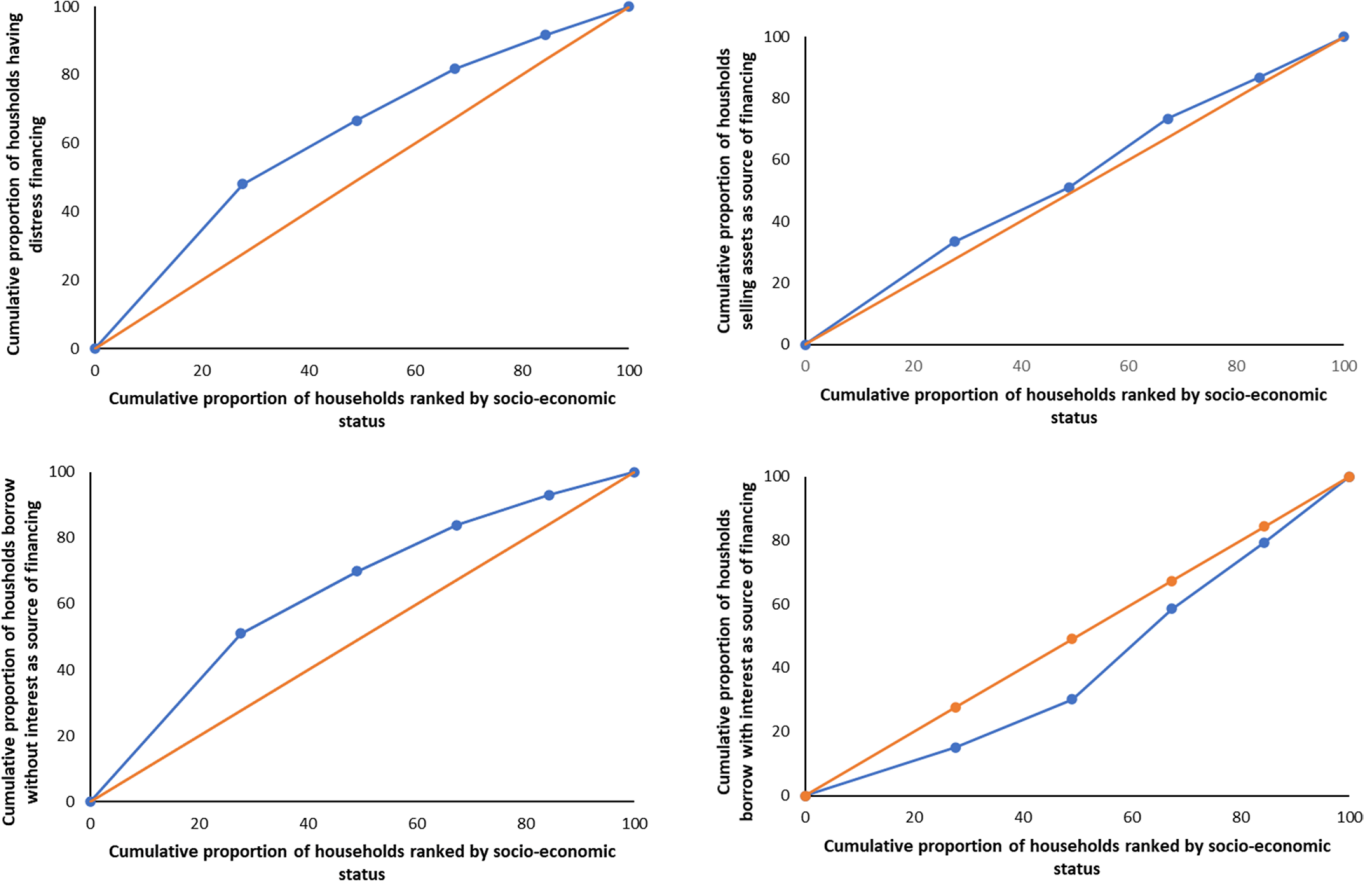
Concentration curves for distress financing and various sources of financing in Malaysia. **A** Concentration curve for distress financing. **B** Concentration curve for Selling assets. **C** Concentration curve for borrowing without interest. **D** Concentration curve for borrowing with interest. The blue line signifies the concentration curve, while the orange line represents the equality line

Figure 2; Table 3 show the concentration curve and concentration index of distress financing and various sources of financing among the socio-economic group. The results revealed that the distribution of distress financing was higher among the poor, with the curve extended above the equality line, portrayed by a concentration index value of -0.245. Borrowing without interest followed the same trend, in which the curve stretched above the equality line with a concentration index value of -0.284. Results also showed that the concentration of household selling assets was slightly higher among the poor, with a concentration index value of -0.065. However, the concentration curve for the prevalence of borrowing with interest fell below the equality line, indicating a higher distribution among the rich. The concentration index value for borrowing with interest was 0.184.

**Table 3:** Concentration index for distress financing and various sources of financing in Malaysia

	Concentration Index	SE
Distress Financing	-0.245	0.018
**Sources of financing**		
Sell of assets	-0.065	0.083
Borrowing without interest	-0.284	0.018
Borrowing with interest	0.184	0.070

Note: SE is Standard Error

### Determinants of distress financing

Table 4 shows the results of the modified Poisson regression model of the distress financing determinants among the households in Malaysia. The model incorporates nine explanatory variables. These variables were the household location, a total of household members aged 65 years and older, a total of household members aged less than five years, household size, socio-economic status, a total of household members who received outpatient care in the past two weeks, a total of household members who received inpatient care in the past 12 months, a total of household members who received oral healthcare in the past 12 months and supplementary health coverage. The model encompassing all the nine explanatory variables was statistically significant, χ^2^ = 347.91, *p* < 0.001. The model explained 4.9% (Cox-Snell R squared) to 8.4% (Nagelkerke R squared) of the variance in distress financing. The results showed that only four variables were statistically significant to determine distress financing, namely the total of household members aged 65 years and older, the total of household members aged less than five years, socio-economic status, and the total of household members who received inpatient care in the past 12 months. Households in poor, middle, rich, and richest socio-economic groups had 0.57, 0.58, 0.40 and 0.36 times the risk to experience distress financing than households in the poorest socio-economic group. The presence of one and two or more household members aged 65 and older were associated with a 1.94 and 1.59 increase in the risk of developing distress financing compared to households with no members aged 65 years and older. The risk of developing distress financing with households having one and two members who received inpatient care in the past 12 months was 1.28 and 1.58 times that of the household with no members who received inpatient care in the past 12 months. Having one household member with an age less than five years also was associated with 0.79 times the risk to develop distress financing than a household without any member aged less than five years.

**Table 4:** Modified Poisson regression model of the distress financing determinants among households in Malaysia (n = 5,146)

Characteristics	β	RR	95% CI for RR	SE for RR	p-value
**Household location**					
Urban (ref.)					
Rural	-0.02	0.98	0.86, 1.13	0.07	0.810
**Total of household members aged 65 years and older**					
0 (ref.)					
1	0.66	1.94	1.68, 2.24	0.14	< 0.001^***^
2 and more	0.46	1.59	1.26, 2.01	0.19	< 0.001^***^
**Total of household members aged less than 5 years**					
0 (ref.)					
1	-0.24	0.79	0.62, 1.00	0.09	0.047^*^
2 and more	0.10	1.11	0.79, 1.55	0.19	0.550
**Household size**					
Less than 5 (ref.)					
5 and more	0.08	1.08	0.87, 1.35	0.12	0.492
**Socio-economic status**					
Poorest (ref.)					
Poor	-0.55	0.57	0.48, 0.69	0.05	< 0.001^***^
Middle	-0.55	0.58	0.47, 0.71	0.06	< 0.001^***^
Rich	-0.91	0.40	0.31, 0.52	0.05	< 0.001^***^
Richest	-1.03	0.36	0.27, 0.48	0.05	< 0.001^***^
**Total of household members received outpatient care in the past 2 weeks**					
0 (ref.)					
1	0.12	1.13	0.96, 1.32	0.09	0.131
2	0.21	1.23	0.92, 1.65	0.19	0.163
More than 2	-0.11	0.90	0.43, 1.86	0.33	0.767
**Total of household members received inpatient care in the past 12 months**					
0 (ref.)					
1	0.24	1.28	1.07, 1.52	0.11	0.006^**^
2	0.46	1.58	1.06, 2.35	0.32	0.026^*^
More than 2	0.09	1.10	0.20, 6.00	0.95	0.915
**Total of household members received oral healthcare in the past 12 months**					
0 (ref.)					
1	0.04	1.04	0.88, 1.22	0.09	0.670
2	-0.15	0.86	0.66, 1.12	0.11	0.256
More than 2	0.16	1.17	0.88, 1.56	0.17	0.288
**Supplementary Health Coverage**					
No coverage (ref.)					
One coverage	-0.08	0.92	0.79, 1.07	0.07	0.288
More than 1 coverage	-0.02	0.98	0.81, 1.19	0.10	0.841

Note: *p < 0.05, **p < 0.01, ***p < 0.001 (significance level at 10%, 5%, and 1%, respectively)

Cox-Snell, R squared = 0.049, Nagelkerke, R squared = 0.084

χ^2^ = 347.91, *p* < 0.001

RR is Relative Risk, CI is Confidence Interval, SE is Standard Error

## Discussions

The present study revealed that the prevalence of borrowing without interest was the highest among various sources of financing at 13.86%, which includes borrowing from friends and family members. Compared to other sources of distress financing, borrowing without interest is considered low risk. Studies also found that selling assets are less common than borrowing since it could push household into poverty [6, 12, 55]. Borrowing is a much more common source of healthcare financing among low- and middle-income countries. However, the prevalence shown in this study was relatively low compared to other countries such as India and Cambodia, of which the prevalence was around 42.2% and 22.5% respectively [19, 48]. The low prevalence of distress financing in Malaysia is aligned with the healthcare financing system in Malaysia, which is mainly tax-based. By subsidising healthcare delivery, the government of Malaysia has provided relatively cheap and universal access to health. The latest analysis showed that Malaysia has a UHC effective coverage index higher than the neighbouring countries such as Indonesia, Myanmar and others [56]. Few initiatives were also implemented to reduce the financial barrier to healthcare such as the PeKa B40, mySalam, *Bantuan Sara Hidup* (BSH) programme, and others which are directly and indirectly cater to the needs of the population to seek healthcare [33].

It is no surprise that the results of the current study showed that the prevalence of distress financing is more concentrated among poor households. This finding is similar to other studies done in Vietnam, Indonesia, India, Myanmar, Nepal, and Ethiopia [11, 57–60]. This current study also found that borrowing without interest was more common among the poor (25.5%) while borrowing with interest was more prevalent among the middle to richest socio-economic groups (1.4–1.6%). The concentration curves and the concentration indices for all sources of financing further explain the differences and unequal distribution of various methods of financing among different socio-economic groups.

Past studies found that households with higher income had a higher level of debt due to confidence in taking a loan [61, 62]. Since access to borrowing without interest greatly depends on social trust, it is more likely to occur among the poor, with the option to borrow with interest being limited [63]. For example, in Southeast India, the ability of a poor household to access borrowing with a low-interest rate depends on their social networks [64]. Notwithstanding the above, the prevalence of borrowing without interest at 13.86% in this current study is almost similar to Cambodia, with 20.8% and 10.9% in 2009 and 2014, respectively [19]. However, the prevalence of borrowing with interest in this current study (1.03%) is much lower compared to Cambodia (69.9%) [19]. Hence, explains the occurrence of inequality among those who borrow with interest. Nevertheless, the prevalence of selling assets was distributed almost equally among the poor and the rich. This is probably due to the low prevalence of selling assets among the households in Malaysia; plus, having no reasons to do so since they have an option to go to public health sectors, which act as the safety net for healthcare delivery in Malaysia. [38].

The presence of family members aged 65 years and older were also associated with the occurrence of distress financing among households. This result is comparable to other studies done in China, India, Cambodia, and Vietnam [65–71]. The presence of the elderly among household members increases the health financial dependency since they are prone to suffer from illnesses and disabilities [72, 73]. Studies have shown that even in high-income countries, the elderly tend to develop distress financing and CHE due to chronic diseases such as diabetes mellitus and cardiovascular diseases [71, 74]. According to NMHS 2019, the elderly contributes to 40.0% of outpatient care and 16.6% of inpatient care utilisation in Malaysia [75].

Analysis of different socio-economic groups revealed that households in the poor socio-economic group were prone to distress financing due to insufficient resources. The socio-economically vulnerable groups, especially the poor, would rely on financing sources such as borrowing and selling assets to get medical treatment [6, 76]. Hence, forcing them to be placed in a very disadvantageous situation and trapped in the vicious cycle of poverty, health, and indebtedness [6].

Spending nights in hospitals can also result in households resorting to distressed sources of financing. This is seemingly related to the increased cost of illness during hospital stays [77]. For example, any illness among family members such as injuries or NCDs, that requires admission will incur an additional economic burden to the respective household, especially when the family’s sole breadwinner is the one affected. This leaves them with limited choices to finance their healthcare costs by either borrowing or selling their assets [78]. Inpatient care is known to cost more to the patient and their family than outpatient care inconsequential either in private or public healthcare facilities [19].

This study also found that the presence of a child aged less than five years old was less likely to results in the household opting for distressed sources of financing. In most countries, maternity and child health services would incur higher costs to the family compared to those who received free-of-charge health services [79]. Perhaps, this additional cost is not significant for a newly formed family especially when both are working parents. Besides, the decreasing trend of mortality and morbidity among children in Malaysia may suggest lower financial demand to care for them [80].

This is the first study looking into the inequality and determinants of households’ financial distress in Malaysia. While determinants of financial distress were the main focus of this study, equality analysis of financial distress and their various sources of financing gives a better understanding of the current financial distress and UHC situation in Malaysia. Rather than using binomial logistic regression, this study adopted Modified Poisson regression to estimate the relative risk. It is the preferable choice of analysis for binomial outcomes in providing unbiased estimates of relative risk when dealing with model misspecification [52]. The causes of model misspecification may include omission of important explanatory (or independent) variables, exclusion of non-linear components or critical interaction terms, or measurement errors [52]. Hence, adopting modified Poisson regression could avoid biased estimates and misleading conclusions. This study also used national household survey data, providing a better representation of the overall financial distress situation among households in Malaysia. The results of this study can be used as a guide to better improve healthcare delivery in Malaysia. While this study revealed that borrowing with interest and selling assets is very low in Malaysia, the prevalence of borrowing without interest was relatively high, especially among the poor socio-economic group and those living in rural areas. Despite the government subsidising the public healthcare sector and providing almost free healthcare services to all citizens, it only covers the costs of treatment and management in hospitals and clinics [24]. Hence, the direct non-medical costs such as transportation costs are still incurred by the patients and their family members. A unique financial initiative such as PeKa B40 which covers the costs of transportation is a good start to improve the situation, but the provision is only limited to the lowest 40% of the income group and the uptake rate was still debatable [81]. Expansion of the programme will ultimately improve the financial distress situation in Malaysia. Particular attention should be given to the elderly, those admitted to the hospitals, and the poor socio-economic group. Since the poor socio-economic groups are more prone to health morbidities, it is imperative that they are being protected financially. Financial distress among the poor would create unequal access to healthcare and subsequently affect their health status. Thus, removing the financial distress could potentially improve their health status and remove the inequalities gap, which is the core to achieving UHC.

Nevertheless, this study is not without limitations. Since this study used cross-sectional data, the results could not provide a causal relationship between independent variables and distress financing. In addition, the trend of financial distress over the years also could not be examined. Changes in financial distress trends are a good indicator to measure the progression of the country’s healthcare systems towards achieving UHC. Using secondary data also limits the analysis to data availability. Variables that are important and necessary but not available could not be incorporated into the analysis [82–84]. Future studies of distress health financing trends will provide a better understanding of the UHC progression in Malaysia and a sustainability gauge for the current healthcare financing systems. In addition, the future study also should explore in-depth reasons and factors behind patients or households resorting to distress financing.

## Conclusions

The prevalence of financial distress in Malaysia can be considered low in Malaysia compared to the neighboring countries reflecting tax-based financial healthcare systems, provision of UHC and additional financial incentives through various government initiatives. Similar to other countries, heavy dependence on financial distress was determined by socio-economic status, the presence of household members aged 65 and older, the presence of household members aged less than five, and the presence of household members who received inpatient care. While the prevalence of financial distress is more concentrated among the poor, the prevalence of much risky behaviour of borrowing with interest was more concentrated towards the rich suggesting a limited source of financing among the poor. The findings presented in this study could pave the way for policymakers to strengthen the healthcare systems by narrowing the socio-economic gap and social security safety nets for a better improvement of healthcare coverage. This study also reiterates that special attention to financial coverage should be given to those households caring for the elderly, requiring admission to hospitals, and lower socio-economic groups.

## Data Availability

The data used and generated in this study is derived from the Healthcare Demand module under NHMS 2019. The data is not publicly available to protect the participants’ privacy. The data may be requested from the corresponding author and Head of Centre for Biostatistics & Data Repository, National Institutes of Health, Ministry of Health Malaysia on reasonable request and with permission from the Director General of Health, Malaysia.

## Abbreviations

B40: bottom 40% of population
BSH: *Bantuan Sara Hidup*
CHE: catastrophic health expenditure
DOSM: Department of Statistics Malaysia
EB: Enumeration Block
EPF: Employee Provident Funds
GDP: Gross Domestic Product.
GEE: generalized estimation equation
LQ: Living Quarter.
MCAR: Missing Completely at Random
MNHA: Malaysia National Health Account
MYR: Malaysian Ringgit
NCD: Non-Communicable Disease
NHMS: National Health and Morbidity Survey
OOP: Out-of-pocket
PeKa B40: *Skim Peduli Kesihatan* for the B40
SOCSO: Social Security Organisation
UHC: Universal Health Coverage
WHO: World Health Organization
